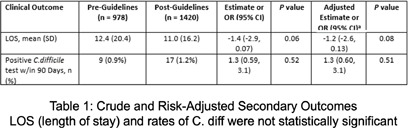# Effect of UTI treatment guideline implementation on antibiotic duration and selection

**DOI:** 10.1017/ash.2025.265

**Published:** 2025-09-24

**Authors:** Jonathan Huang, Sujit Suchindran, Benjamin Albrecht, Sarah Green, Julianne Kubes, Lucy Witt

**Affiliations:** 1 Emory University School of Medicine; 2 Emory University School of Medicine; 3 Emory University Hospital; 4 Emory University Hospital; 5 Emory Healthcare; 6 Emory University

## Abstract

**Background:** Clinicians have variable prescribing practices for treating urinary tract infections (UTI), resulting in broader and longer treatment durations than necessary. In March 2023, guidelines for UTI treatment were developed and disseminated across our hospital system. **Methods:** We evaluated inpatients at Emory University Hospital (EUH) who received antibiotics with an indication of UTI between November 2022 and March 2024 to investigate implementation effect on treatment duration and choice. We characterized days of therapy (DOT) by performing interrupted time series analysis, adjusting for demographic and clinical variables. Additionally, we looked at percent use of guideline concordant antibiotics chosen before and after implementation. **Results:** A total of 978 cases of UTIs were evaluated pre-guideline implementation among 621 (63.5%) females with 490 (50.1%) Black patients. A total of 1420 cases of UTIs were evaluated post-guideline implementation among 843 (59.4%) females with 693 (48.8%) Black patients. With inpatient UTI DOT, following implementation there was an increase by 31.5 DOT/month with a sustained increase by 6.25 DOT/month with no statistically significant change. Total UTI DOT (including outpatient) showed a sustained decrease by 21.1 DOT/month with no overall statistical significance. With inpatient UTI guideline concordance, following implementation there was an increase by 2.5% per month with a sustained increase by 0.7% per month with no statistically significant change. Total UTI guideline concordance (including outpatient) showed a sustained increase by 1.4% per month with no overall statistical significance. **Conclusion:** Guideline implementation for UTI treatment did not lead to statically significant change in DOT or guideline concordant prescribing at EUH.

**Figure:**
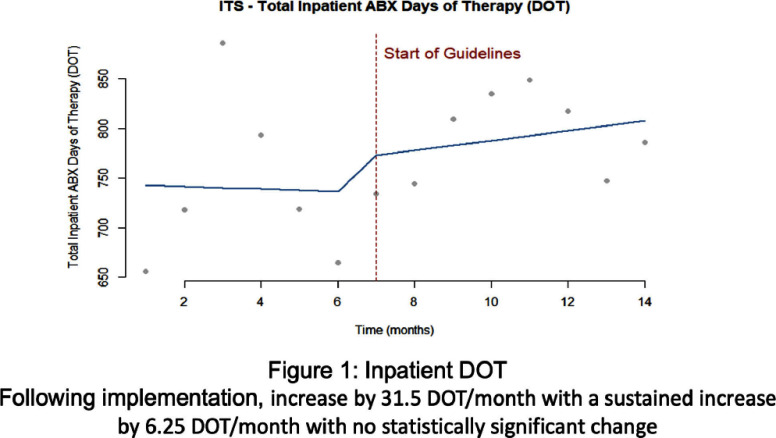

**Figure:**
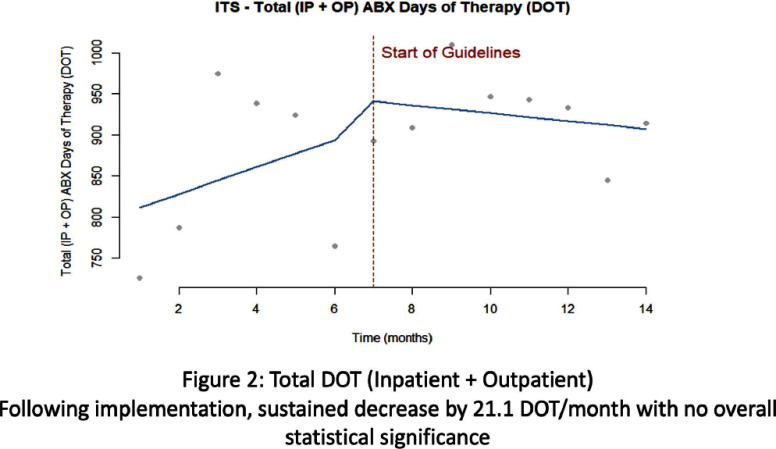

**Figure:**
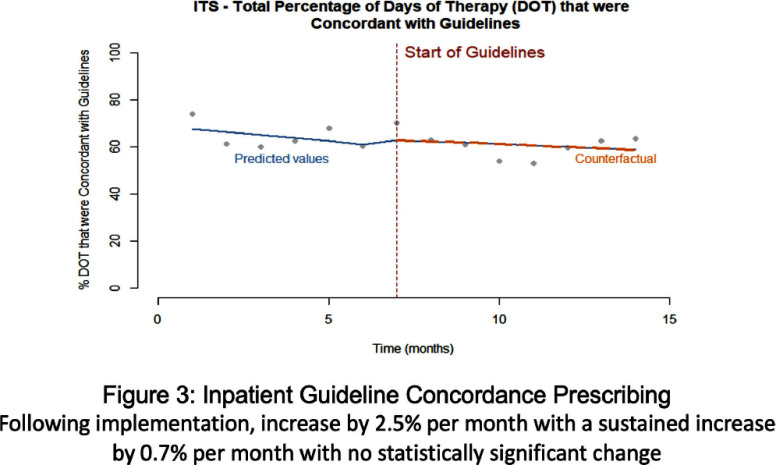

**Figure:**